# The Effect of Young Age in Hormone Receptor Positive Breast Cancer

**DOI:** 10.1155/2015/325715

**Published:** 2015-08-16

**Authors:** Minna K. Lee, Leo A. Varzi, Debra U. Chung, Minh-an Cao, Jeffrey Gornbein, Sophia K. Apple, Helena R. Chang

**Affiliations:** ^1^University of California, Los Angeles, Los Angeles, CA 90095, USA; ^2^Department of Surgery, David Geffen School of Medicine, Los Angeles, CA 90095, USA; ^3^Revlon/UCLA Breast Center, Los Angeles, CA 90095, USA; ^4^Department of Biomathematics, David Geffen School of Medicine, Los Angeles, CA 90095, USA; ^5^Department of Pathology, David Geffen School of Medicine, Los Angeles, CA 90095, USA

## Abstract

*Background*. Studies have shown that young breast cancer patients have more advanced disease and worse survival compared to older patients. Our objective was to study disease characteristics and survival in the subset of young women with hormone receptor positive (HR+) and HER2 negative (HER2−) cancer. *Methods*. We retrospectively analyzed HR+/HER2− breast cancer patients who underwent surgery at our institution between 2002 and 2010. We compared clinical characteristics, pathology, treatment, and recurrence-free survival between younger (≤40 years) and older (>40 years) patients. *Results*. Of 669 HR+/HER2− breast cancer cases, 54 (8.1%) patients were 40 years or younger. Younger patients had more luminal B subtype, high grade, poor differentiation, and increased lymphovascular invasion. Younger women were treated more often with mastectomy and adjuvant chemotherapy. Although the unadjusted recurrence-free survival at median 55-month follow-up was lower in younger women, adjusting for stage, there was no significant difference (90.7% versus 89.3%, *p* = 0.74) between groups. *Conclusion*. Younger patients with HR+/HER2− breast cancer had more advanced disease and more aggressive treatment than older patients. The unfavorable pathologic features suggest a biologically different tumor in young women. After adjusting for these factors, younger patients have a recurrence-free survival similar to older patients.

## 1. Introduction

Although the incidence of breast cancer in young women is low, it is the leading cancer-related death in women younger than 45 years of age. Young women in this age group account for approximately 11% of new breast cancer cases and approximately 6% of breast cancer-related deaths [1]. The question of whether young age alone is an independent prognosticator in breast cancer patients has been in debate. Several studies have reported that breast cancer in young women is associated with worse outcomes including higher mortality and recurrence rates compared to older women [2–6]. However, many others have shown that young age is not an independent predictor of poor survival when controlling for other confounding factors [7–9].

Reports have found that breast cancer in young women is associated with advanced stage of disease and unfavorable tumors characteristics frequently attributed to the higher frequency of more aggressive subtypes of breast cancer such as triple-negative breast cancer (TNBC) and HER2 positive (Her2+) breast cancer [10, 11]. The objective of this study was to exclude the more aggressive subtypes of breast cancer and only examine whether young age affects hormone receptor positive (HR+) and HER2 negative (HER−) breast cancer. The knowledge of an age-specific breast cancer even within this most favorable subtype of breast cancer may help to tailor a more age directed treatment since the outcome of young women's cancer is likely to be determined by both the biology of the tumor and the appropriately chosen treatment.

## 2. Patients and Methods

**Figure 1:**
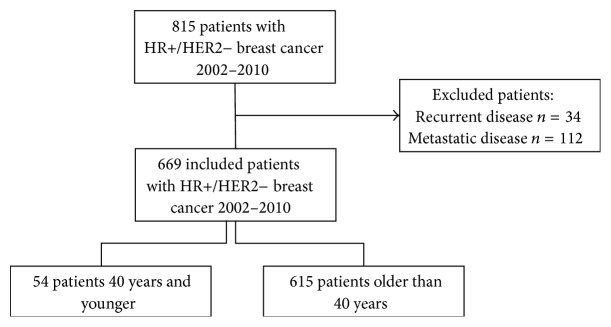
Schema of inclusion and exclusion criteria.

We performed a retrospective review of women with HR+/HER2− breast cancer who were treated at our breast center between 2002 and 2010. After approval by the institutional review board (IRB) and in compliance with Health Insurance Portability and Accountability Act (HIPAA) regulations, this study included all patients with tissue diagnosis proven HR+/HER2− breast cancer. Patients with recurrent or metastatic disease at the time of their initial presentation were excluded (Figure 1). Patients were separated into two groups based on their age at first diagnosis. The young group included women 40 years of age and younger and the comparison group included women older than 40 years of age. The pathology, type of surgery, and postoperative treatment regimens were compared between groups. Follow-up, recurrence, and survival information was gathered from postoperative follow-up notes with surgeons, medical oncologist, and radiation oncologists as available. As our institution is a tertiary care center, many patients travel long distances for their surgical management and then receive their adjuvant treatment locally. Therefore, adjuvant hormonal therapy data was unobtainable for most of our cohort and not included in this analysis. At our institution between 2002 and 2010, patients of Ashkenazi Jewish descent were tested for the founder mutation. All other patients underwent full sequencing for BRCA 1 or 2 mutations. Last known follow-up was considered as the most recent date the patient was entered into the electronic medical record system for our institution.

### 2.1. Pathology

All patients underwent surgical management at our institution; therefore, the same team of breast pathologists reviewed all tumor samples. Pathological variables included receptor status, Her-2/neu status, Ki-67, histological grade, modified Bloom and Richardson score and differentiation, and lymphovascular invasion. HER2 by immunohistochemical (IHC) staining was scored as follows: 0 with no staining observed or membrane staining that was incomplete and was faint/barely perceptible and within less than or equal to 10% of tumor cells, 1+ with incomplete membrane staining that was faint/barely perceptible and within greater than 10% of tumor cells, 2+ with circumferential membrane staining that is incomplete and/or weak/moderate within greater than 10% of tumor cells or complete and circumferential membrane staining that is intense and within less than or equal to 10% of tumor cells, and 3+ with circumferential membrane staining that is complete, intense, and within greater than 10% of tumor cells. IHC 0 and IHC 1+ were considered to have no overexpression. IHC 2+ was equivocal, and IHC 3+ was considered to be overexpression. HER2 was defined by fluorescent in situ hybridization (FISH) as nonamplified when dual-probe HER2/CEP17 ratio was less than 2.0 and amplified when dual-probe HER2/CEP17 ratio was greater than or equal to 2.0. Although the recent guidelines for diagnosing HER2 positive disease have been revised, we elected to use a HER2/CEP17 ratio greater than or equal to 2.0 for the threshold of amplification as this was the guideline during the time our patient population was diagnosed. For analysis of estrogen receptor (ER) and progesterone receptor (PR) status, tumors with greater than or equal to 1% of cell nuclei stained were considered positive, regardless of staining intensity, respectively. For analysis of Ki-67, IHC was performed on 4-micron-thick sections of formalin-fixed paraffin-embedded tumor tissue using Ki-67 antibody M1B1 (Dako, Carpinteria, CA) in 1 : 100 dilution with 1 : 600 dilution in phosphate-buffered saline. Detection system and machine used were DAKO and Leica-Bond autostainer. Proliferation index was considered high if IHC staining for Ki-67 was greater than 14%. In accordance with the St. Gallen International Expert Consensus of 2011, tumors were considered luminal A subtype if Ki-67 was less than 14% and luminal B subtype if Ki-67 was greater than or equal to 14% [12]. Staging followed criteria of the American Joint Commission on Cancer Manual for Staging of Cancer [13].

### 2.2. Statistical Analysis

The *p* values for comparing means of continuous variables that followed the normal distribution were computed using a one-way analysis of variance model. Otherwise, *p* values for comparing continuous or ordinal variables across groups were computed using the Kruskal-Wallis test. Categorical variables were compared using Fisher's exact test or the chi-square test as appropriate. Recurrence-free survival was calculated from the date of initial diagnosis through the date of last follow-up. Survival probabilities and durations were estimated using the Kaplan-Meier method. Survival curves were compared using the log rank test. The simultaneous effect of potential predictors including age on recurrence-free survival was assessed using the multivariate Cox proportional hazards model. Significant variables were chosen under this model using stepwise backward selection and a liberal *p* < 0.10 variable retention criterion. Hazard rate ratios (HRs) based on this model are reported along with their 95% confidence bounds and *p* values for comparison with HR = 1. Statistical analyses were conducted using SAS version 9.4 (SAS Inc., Cary, NC).

## 3. Results

Of 669 cases of breast cancer analyzed, 54 (8.1%) were in women 40 years old and younger. There was no difference in the incidence of a family history of breast cancer between groups (44.4% versus 38.7%, *p* = 0.51). Young women were more likely to undergo BRCA1/2 testing (48.1% versus 12.0%, *p* < 0.01) and, of those tested, were more likely to be positive. Young women had tumors with higher grade (50.0% versus 22.1%, *p* < 0.01), poor differentiation (37.0% versus 22.1%, *p* < 0.01), and lymphovascular invasion (37.0% versus 22.1%, *p* = 0.04) compared to older women. Although there was no difference in the percent of ER+ cases between groups, younger women showed a decreased median of positive ER staining (80% versus 90%, *p* < 0.01) compared to older women. Young women also had increased rates of luminal B subtype (63.0% versus 41.8%, *p* < 0.01). There was no difference in tumor (T) stage between younger and older women; however, younger women were more likely to have N1 (37.0% versus 25.2%) and N2 (16.7% versus 5.9%, *p* < 0.01) disease. Young women trended to have more stage II (50.0% versus 42.1%) and stage III (22.2% versus 14.6%, *p* = 0.07) disease compared to older women (Table 1).

Compared to older women, young women were more likely to undergo mastectomy (75.9% versus 47.5%, *p* > 0.01) and axillary lymph node dissection (55.6% versus 42.6%, *p* = 0.05). Twenty-two (44%) of younger women also had a prophylactic mastectomy on the contralateral side. For adjuvant treatment, younger women were more likely to receive chemotherapy (53.7% versus 37.6%, *p* < 0.01) compared to older women although there was no difference in the rates of neoadjuvant treatment (9.3% versus, 6.0%, *p* = 0.43). There was no difference in the rates of postoperative radiation therapy (Table 2).

**Figure 2:**
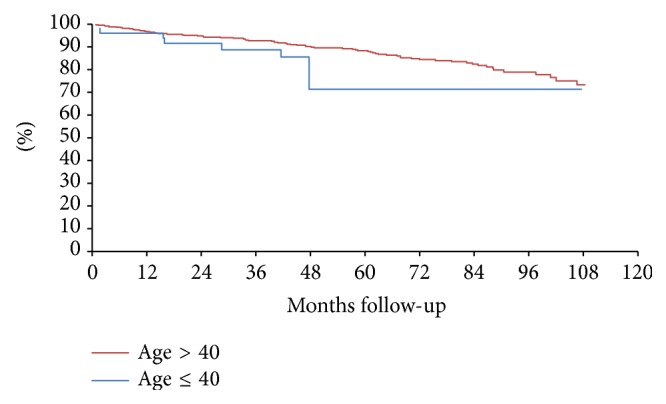
Unadjusted recurrence-free survival at median 55-month follow-up (83.3% versus 89.9%, HR = 0.92, *p* = 0.83).

**Figure 3:**
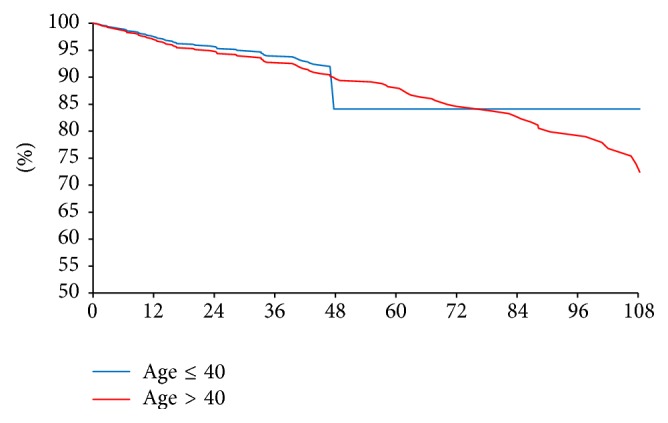
Recurrence-free survival adjusted for stage (90.7% versus 89.3%, HR = 1.16, *p* = 0.74).

Median follow-up time for younger women was 49 months and for older women was 53 months. Overall median survival for the entire cohort was 53 months. The unadjusted recurrence-free survival at median 55-month follow-up was lower in younger women (83.3% versus 89.9%, HR = 0.92, *p* = 0.83) as seen in Figure 2. After adjusting for stage, there was no significant difference between groups (90.7% versus 89.3%, HR = 1.16, *p* = 0.74) as seen in Figure 3. In multivariate Cox regression analysis, higher stage, luminal B subtype, breast conservation treatment, lack of adjuvant chemotherapy, and postoperative radiation were independent risk factors for recurrence or death. Controlling for these factors as well as tumor and nodal stage, tumor grade and differentiation, presence of lymphovascular invasion, and nodal surgery, young age alone was not a risk factor in patients with HR+/Her2− breast cancer (HR = 1.10, *p* = 0.84, Table 3).

## 4. Discussion

Although breast cancer in young women is uncommon, it has received attention due to its association with unfavorable outcomes. Young age itself has been reported as an independent risk factor for recurrence and death [2–6, 11]; however, many of these studies are decades old, have included patients with all subtypes of breast cancer, and may not have adequately controlled other risk factors. A main limitation of these studies is that different subtypes may be more prevalent in young women, which may have varying prognostic and treatment implications [10, 14]. The aim of this study was to investigate the effect of young age on tumor pathology, multidisciplinary treatment regimens, and recurrence-free survival limited to a breast cancer subtype with HR+/HER2− expression where other risk factors were controlled.

Our study found that young patients with HR+/HER2− breast cancer had more advanced disease associated with less favorable histopathologic features. Women 40 years old and younger were more likely to have high grade, poorly differentiated tumors, and lymphovascular invasion. More young women in our series also had higher stage of disease. These results are consistent with findings in the literature. Interestingly, our study also suggested that young women had increased rates of luminal B HR+/HER2− breast cancer. Luminal B breast cancer is associated with a more aggressive disease phenotype and worse outcomes as compared to luminal A cancers [15]. Maggard et al. [16], Kheirelseid et al. [17], and Gnerlich et al. [18] found that younger breast cancer patients, inclusive of all subtypes, had higher grade tumors and presented with later stage disease. Sidoni et al. showed that women under 40 years had a more aggressive tumor profile than older patients and that carcinomas in younger women were characterized by genetic instability [19]. Taken together, these results suggest that the underlying tumor biology in many of these younger patients has a more aggressive profile and less favorable outcome. Our study indicates that young patients tend to have a biologically more aggressive phenotype even within the more favorable HR+/HER2− breast cancer subtype.

Given the evidence that younger women have more aggressive tumor biology such as TNBC or HER2+ subtypes of breast cancer, it is understandable to expect an unfavorable outcome in this group of women. In this series, we focused our questions on young women with luminal breast cancer, a non-TNBC and non-HER2+ breast cancer. Even in this most favorable subtype of breast cancer, we found that young women were more likely to undergo more aggressive breast cancer surgery such as mastectomy and axillary lymph node dissection due to more advanced stage of disease at the time of diagnosis. Livi et al. [20] examined women aged less than 35 years and found that surgical treatment was not a predictor of local recurrence. On the contrary, Gajdos et al. [9] and Anderson et al. [21] found higher rates of local recurrence in young women treated with breast conservation as compared with mastectomy. Younger women in our study were more likely to be BRCA 1 or 2 positive likely leading to a high rate of both mastectomy and double mastectomies seen in this group. Our study also showed that young patients were also more likely to receive adjuvant chemotherapy. This finding is consistent with others who reported that adjuvant chemotherapy benefits young women in both reducing recurrence rates and increasing overall survival [22, 23].

Studies comparing survival between young women and older women have produced conflicting results. Xiong et al. found that young patients less than or equal to 30 years had poorer overall survival compared to older patients [4]. Likewise, Dubsky et al. examined young women 35 years old compared to women older than 35 years and found that young age was a powerful independent prognostic factor in multivariate analysis of recurrence-free and overall survival [6]. However, many others had different conclusions [9, 17, 18]. In this series, when controlling for confounding variables, age alone was not a predictor of decreased recurrence-free survival. Multivariate analysis showed that increased stage, luminal B subtype, breast conservation treatment, and lack of adjuvant chemotherapy and radiation were risk factors for recurrence and death.

Limitations of this study included its retrospective nature with small sample size and a relatively short median follow-up of 55 months. As a single institution study at a tertiary care center, the cohort presented may not represent the general population as a whole. Although variation in patient management over time, particularly in multicenter studies, is a potential bias in any retrospective study, this effect is minimal in this report since the same breast oncology team treated patients in the recent years. A larger sample size with more complete data as well as longer follow-up time may reveal other clinically relevant differences between young women and older women with HR+/HER2− breast cancer. Nonetheless, our data suggest, in agreement with the current literature, that young age alone is not a risk factor for worse outcomes in HR+/HER2− breast cancer.

## 5. Conclusions

In this study, we found that younger patients with HR+/HER2− breast cancer have more advanced disease and are treated more aggressively than older patients. Many unfavorable histopathologic characteristics of the tumors were found to be associated with young patients including higher grade and increased prevalence of luminal B subtype which suggests that young women may have different tumor biology. However, after adjusting for these factors, stage of disease, and treatment, there was no difference in recurrence-free survival between younger and older patients.

## Figures and Tables

**Table 1:** Preoperative and pathologic characteristics in young women compared to older women.

Variable	≤40 years (*n* = 54)	>40 years (*n* = 615)	*p* value
Age, median (IQR)	36 (32–39)	58 (50–67)	0.00
Breast cancer family history (%)	24 (44.4)	238 (38.7)	0.51
BRCA1/2 tested (%)	26 (48.1)	74 (12.0)	<0.01
BRCA1/2 positive	11	13	0.02
Neoadjuvant treatment (%)	5 (9.3)	37 (6.0)	0.43
Grade (%)			
Low	2 (3.7)	130 (21.1)	<0.01
Intermediate	24 (44.4)	345 (56.1)	
High	27 (50.0)	136 (22.1)	
Differentiation (%)			
Well	7 (13.0)	237 (38.7)	<0.01
Moderate	27 (50.0)	262 (42.6)	
Poor	20 (37.0)	105 (17.1)	
Lymphovascular invasion (%)	20 (37.0)	136 (22.1)	0.04
Receptor status			
ER+ (%)	52 (96.3)	609 (99.0)	0.13
ER% staining, median (IQR)	80 (60–95)	90 (80–95)	<0.01
PR+ (%)	45 (83.3)	514 (83.6)	0.99
PR% staining, median (IQR)	55 (10–90)	60 (10–90)	0.92
Ki67, median (IQR)	12 (7–17)	12 (7–17)	0.99
Luminal subtype (%)			
Luminal A	20 (37.0)	358 (58.2)	<0.01
Luminal B	34 (63.0)	257 (41.8)	
Tumor stage (%)			
T1	25 (46.3)	329 (53.5)	0.55
T2	22 (40.7)	205 (33.3)	
T3	6 (11.1)	56 (9.1)	
T4	1 (1.9)	25 (4.1)	
Nodal stage (%)			
N0	25 (46.3)	375 (61.0)	<0.01
N1	20 (37.0)	155 (25.2)	
N2	9 (16.7)	36 (5.9)	
N3	0 (0)	21 (3.4)	
Stage (%)			
I	15 (27.8)	266 (43.3)	0.07
II	27 (50.0)	259 (42.1)	
III	12 (22.2)	90 (14.6)	

ER: estrogen receptor.

PR: progesterone receptor.

IQR: interquartile range.

**Table 2:** Types of treatment in young women compared to older women.

Variable	≤40 years (*n* = 54)	>40 years (*n* = 615)	*p* value
Surgery (%)			
Breast conservation	13 (24.1)	323 (52.5)	<0.01
Mastectomy	41 (75.9)	292 (47.5)	
Nodal treatment (%)			
None	0 (0)	41 (6.7)	0.05
SLND	24 (44.4)	312 (50.7)	
ALND	30 (55.6)	262 (42.6)	
Adjuvant treatment (%)			
Chemotherapy	29 (53.7)	231 (37.6)	<0.01
Radiation therapy	24 (44.4)	350 (57.3)	0.18

SLND: sentinel lymph node dissection.

ALND: axillary lymph node dissection.

**Table 3:** Cox proportional hazards model for recurrence/death.

Variable	Hazard ratio	95% confidence interval	*p* value
Age <40 years	1.10	0.46, 2.63	0.84
Stage			
2 versus 1	1.90	0.80, 4.54	0.15
3 versus 1	8.93	2.93, 27.21	<0.01
Luminal B	1.93	1.16, 3.22	0.01
Surgery			
Mastectomy versus BCT	0.42	0.24, 0.74	<0.01
Postoperative chemotherapy	0.40	0.23, 0.67	<0.01
Postoperative radiation	0.31	0.18, 0.54	<0.01

BCT: breast conservation therapy.

Nodal stage, differentiation, lymphovascular invasion, nodal surgery, and T stage not significant at *p* < 0.05 given the above model.
